# Localization of Impacted Canines - A Comparative Study of Computed Tomography and Orthopantomography

**DOI:** 10.25122/jml-2020-0001

**Published:** 2020

**Authors:** Asadullah Khan Mohammed, Garepally Sravani, Deepti Vallappareddy, Annamaneni Rakesh Rao, Arshad Qureshi, Anisha N Prasad

**Affiliations:** 1. Department Of Orthodontics, Sri Balaji Dental College, Hyderabad, Telangana, India; 2. Department Of Orthodontics, Sri Sai College Of Dental Surgery, Vikarabad. Telangana, India

**Keywords:** Computed Tomography, Diagnosis, Impacted Canine, Orthodontist, Orthopantomograph

## Abstract

Our aim was to evaluate the location of impacted canines and their proximity to the lateral and central incisor to assess the prognosis of the impacted canine and compare the reliability of 2D versus 3D imaging solutions.

We carried a prospective study on 17 subjects with impacted maxillary canines. Later, the patients underwent radiologic examination, i.e., sectional computed tomography and orthopantomography. The obtained records were compared regarding the location of the impacted maxillary canine, the proximity and resorption of the impacted canine to the lateral and central incisors, the prognosis of the impacted canine, and the linear distance of the canine from the vertical and horizontal reference lines drawn.

Eleven males (45.8%) and 13 females (54.1%), 11 (45.8%) impacted canines on the right side, and 13 (54.1%) on the left side, as well as 2 (8.3%) transpositions, were noted. Buccally impacted teeth caused less resorption of the adjacent teeth. Among the 24 teeth examined using orthopantomography with the sector method, 3 (12.5%) teeth were found in sector 1. There were 8 (33.33%) teeth in sector 2, and 11 (45.8%) of the impacted canines were in sector 3. In localizing impacted maxillary canines, computed tomography revealed an increased accuracy of 31% compared to orthopantomography.

Even though both computed tomography and orthopantomography revealed similar records, computed tomography showed more accuracy, also having an added advantage of its 3D viewing capabilities in precise localization of the impacted maxillary canine.

## Introduction

The term impaction is defined as a lack of eruption of a tooth within the physiological limits of time of the normal eruption process. Canines are the second most frequently impacted teeth after third molars, with a prevalence ranging from 0.92% to 4.3% [2]. An accurate diagnosis of the accurate location of the impacted canine and the surrounding structures is necessary to manage the pathologic condition properly [1, 2].

Impacted canines have a multi-factorial etiology, including genetic origin, lateral incisor obstruction, and others. Buccal canine impaction is usually due to insufficient space and crowding, whereas canine palatal impaction is thought to be due to the presence of excess space in the upper arch, which might make the canine to cross back from the buccal to the palatal side [2-4].

The management of impacted canine requires a multidisciplinary approach comprising of a pediatric dentist, oral surgeon, and orthodontist. The major problem in diagnosing impacted canines by means of conventional radiographic methods is the superimposition of structures on the film, making it difficult to distinguish details. Recently, computed tomographic (CT) scanning has been used to provide more reliable details than conventional methods [1, 3].

From an orthodontist's point of view, managing impacted canines require a knowledge of the exact location, based on which a decision will be taken; the tooth should either be exposed, extracted, recovered or left untreated. Various radiographic methods employed for diagnosing impacted canines are orthopantomography (OPG), laterolateral and posteroanterior teleradiography, the parallax method, laterolateral, occlusal radiography, computerized axial tomography, cone beam, and 3D CT elaboration such as 3D rendering. Also, real 3D stereolithographic models can be generated. New imaging techniques such as cone-beam computed tomography (CBCT), having advantages of low radiation, rapid image scanning with radiographic and 3D volumetric data has been used to diagnose and treat patients with impacted teeth [3-5].

Maxillary canines have a vital role concerning mastication, aesthetics, and occlusion. Hence, it is imperative that dental surgeons should correctly diagnose and manage impacted canines [4, 5]. Keeping this need in mind, this study was carried out to compare OPG vs. CT in the localization of maxillary impacted canines, its proximity to the lateral and central incisor, resorption of the adjacent tooth, and its prognosis.

## Material and Methods

This prospective study consisted of a total of 17 subjects with impacted maxillary canines, out of which 7 subjects had bilateral and 10 had unilateral impacted canines, with a total number of 24 impacted teeth. The ages of the patients included in the study ranged from 14 to 23 years, with 11 male and 6 female subjects. The study was carried out between July 2016 to June 2018, after receiving approval from the ethics committee (ECR/SSCDS.12/2016) and written informed consent was obtained from all subjects. The sample size was calculated using the G power software. The total sample size obtained was 17.

### Inclusion Criteria:

1. All the patients should have permanent dentition;
2. The patient should have a minimum age of 14 years old;
3. Only maxillary canine impactions were considered;
4. Both unilaterally and bilaterally impacted canines were considered.

In 65% of cases, over-retained deciduous canines were present. Over retention was determined by the patient's chronological age and the average age in which the deciduous canine exfoliates is 11 years ± 9 months for maxillary teeth.

### Exclusion Criteria:

1. Patients who only had deciduous dentition;
2. Patients who have been treated orthodontically prior to the study;
3. Patients with syndromes or systemic diseases that are known to affect eruption pattern significantly;
4. At the time of our protocol submission, we decided to include children above 14 years. Therefore, all patients younger than 14 years old were excluded.

Later, the patients underwent radiologic examination, i.e., sectional CT and OPG. The diagnostic records were obtained with informed consent and were compared later. The dental sectional CT machine (SOMATOM™ 64, Siemens, Germany) with a slice thickness of 0.75mm (slice) and the difference between the slices (pitch) of 0.75 × 0.9 mm and image time of 10.7 seconds with a rotation time of 0.33 second was used. With 14.10 mGy, it has 90 mAs of x-ray speed and 120 kV of radiation force, giving a 180 FoV.

The DIACOM™ software allows the valuation and measurement in all three planes of view – axial, coronal, and sagittal view in a CT scan. The perpendicular reference lines were drawn; the one from the midline of the maxilla running between the central incisors until the last erupted molar, and the other reference line was the occlusal line. An angle was constructed by the lines drawn with respect to the occlusal reference line, and the line drawn from the long axis of the impacted canine was used to assess the favorability of the eruption path.

The imaging data were analyzed using the DIACOM software to assess the location of the impacted maxillary canine, the proximity and resorption of the impacted canine to the lateral and central incisors, the prognosis of the impacted canine and the linear distance of canine from the vertical and horizontal reference lines.

In the axial view, a vertical line bisecting the midline of the maxilla was drawn as the reference line. In this view, the location of the canine, whether it is palatal or buccally placed, was assessed. The location of the tip of the canine showed its position in the maxilla.

In a coronal view, two reference lines that consist of a horizontal occlusal plane line and a vertical line bisecting the midline of the jaws were created. The distance from the canine cusp to the vertical reference line was drawn by a perpendicular line, showing its distance from the central reference line. Another perpendicular line was drawn from the canine cusp to the horizontal occlusal reference line, showing its linear distance of the canine to the occlusal surface.

In the sagittal view, a horizontal reference line was created at the occlusal level. From the long axis of the canine, a vertical line was drawn to the horizontal reference line. The angle formed by these two lines was used to assess the angulation of the impacted canine to the occlusal surface.

**Figure 1:**
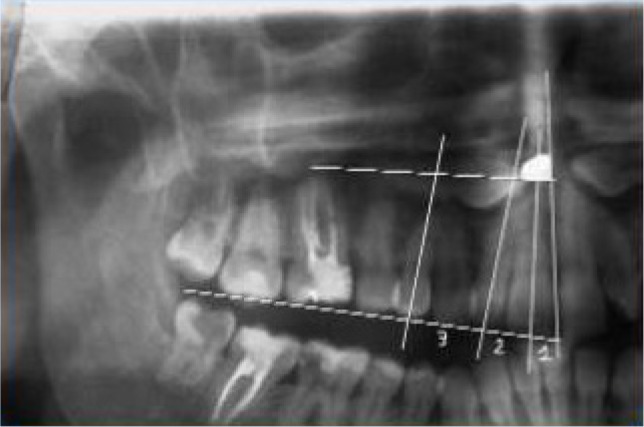
Sector method showing sectors 1, 2 and 3.

The OPG machine (Sirona – Orthophos™ XG5), having a radiation range between 75 and 90 kV (depending on the patient age), with 14.1 second rotation time, was used for recording an OPG test of the subjects. The exposure times ranged from 10 – 16 seconds, depending on the age of the patient. Sidexis® software was used for digitizing the image for recording it on the film. Localization of impacted canine on an OPG was done via the sector method. The long axis of the central incisor, the lateral, and the first bicuspid marked three different areas. The position of the cusp of the canine in each of these sectors suggests their prognostic values (Figure 1).

• Sector 1: If the cusp tip of the canine was between the inter-incisor median line and the long axis of the central incisor.
• Sector 2: If the cusp tip of the canine was between the major axis of the lateral and central incisor.
• Sector 3: If the cusp tip of the canine was between the major axis of the lateral and the first premolar.

An angle “α” (Figure 1) is used to represent the angle formed between the long axis of the impacted canine and inter – incisor median line, and “d” as the perpendicular distance of the peak of the cusp tip of the impacted canine with respect to the occlusal plane.

### Statistical analysis

The analysis was done to compare the observations from the sectional CT and OPG. All the statistical analyses were performed using SPSS version 20 (IBM SPSS Statistics for Windows, IBM Corp., Armonk, NY, USA). Mean comparisons among the subjects for linear measurements of OPG and CT were made using an independent sample t-test. Correlation of various linear measurements of OPG and CT was done using Pearson's correlation coefficient. A p-value of <0.05 was considered statistically significant.

## Results

The distribution of canine impaction among these subjects was 11 males (45.8%) and 13 females (54.1%), 11 (45.8%) impacted canines on the right side, and 13 (54.1%) on the left side; 2 (8.3%) transpositions were also noted (Table 1).

**Table 1:** Incidences of right, left canines and transpositions.

Canine	No. of teeth (24)	% of teeth
Right Canine	11	45.8%
Left Canine	13	54.1%
Transpositions	2	8.3%

Owing to the location of the impacted teeth, 4 (16%) of the lateral incisors, and 1(4%) central incisor were resorbed. In the patient with central incisor resorption, the lateral incisor had resorbed too. The impacted teeth placed buccally caused less resorption of the adjacent teeth, and the impacted teeth on the palatal side had more propensity of resorbing adjacent teeth (Table 2).

**Table 2:** Incisor resorption associated with 24 impacted canines.

Incisors	Resorption	% of Resorption
Lateral incisor	1	4%
Central incisor	4	16%

Of the impacted canines, 9 had proximity with lateral incisors (37.5%) and 7 (29.1%) to the central incisor (Table 3). The impacted canines which were in transposition were in proximity with first and second premolars. This includes one case in which lateral incisors were missing bilaterally. The proximity with the central incisor with buccally placed impacted canines was found in 3 (12.5%) patients; the same values (3 patients, 12.5%) were found regarding the lateral incisor. The proximity of the central incisor with a palatally impacted canine was 4 (16.6%) and with a lateral was 6 (25%). The incidence of buccally placed impacted canine was higher when compared to palatally placed impacted canine.

**Table 3:** Distribution of impacted teeth in different sectors.

Sector	No. of teeth	% of teeth
Sector 1	3	12.5%
Sector 2	8	33.33%

**Figure 2:**
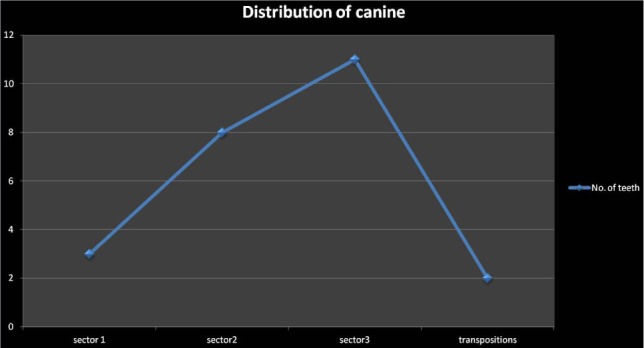
Distribution of canines.

On the OPG examination among 24 teeth examined with the sector method, 3 (12.5%) teeth were found in sector 1. There were 8 (33.33%) teeth in sector 2, and 11 (45.8%) of the impacted canines were in sector 3 (Table 4). The prevalence rate of maxillary impacted canine in sector 3 is greater than sector 2, sector 1, and transpositions (Figure 2).

**Table 4:** Distribution of impacted teeth in different sectors.

Sector	No. of teeth	% of teeth
Sector 1	3	12.5%
Sector 2	8	33.33%
Sector 3	11	45.8%

**Figure 3:**
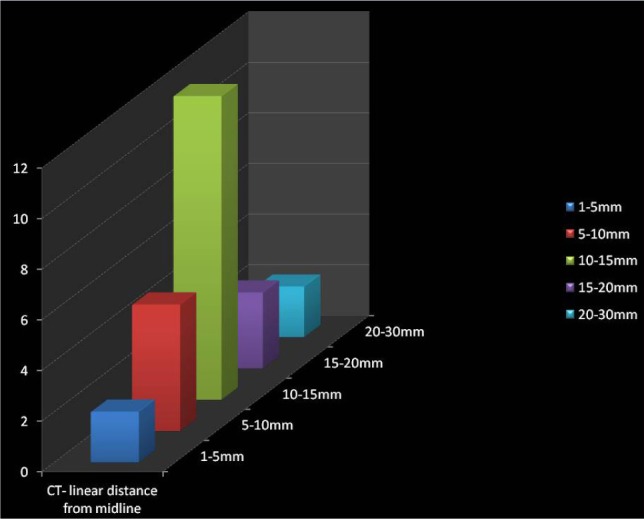
Linear distance from the midline to the cusp of the canine in a CT scan.

**Figure 4:**
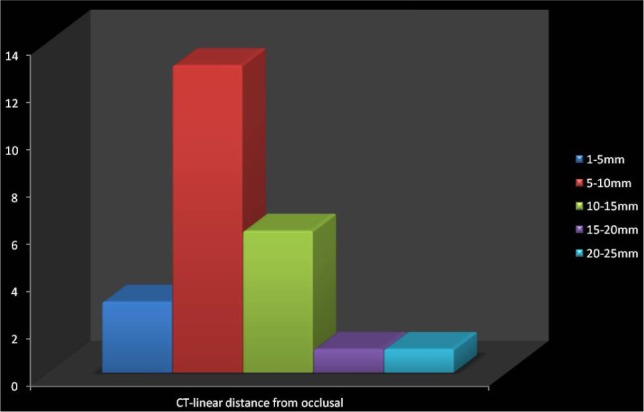
Linear distance from the occlusal plane to the cusp of the impacted canine in a CT scan.

The observations from the CT scans were noted in 3 views:

1. Axial View – The location of the impacted canine was observed. The buccally placed impacted canine was far from the midline compared to the palatally placed impacted canine. It has been observed that there was no resorption of adjacent teeth when the impacted canine was in sector 3 and that there was no proximity to the lateral and central incisors when the impacted canine was in sector 3. Depending upon the location of the impacted canine i.e., if the canine was palatally impacted, its probability of resorbing the lateral and central incisor was higher.
2. Coronal view – The linear distance from the occlusal and mid reference lines were observed. In the buccally placed impacted canine, the mean linear value from the midline was 14.4 ± 2 mm in sector 3, 12 ± 2 mm in sector 2, and 6 ± 1 mm in sector 1. In the palatally placed impacted canine, the mean linear value from the midline was 10 ± 1 mm in sector 3, 6 ± 1mm in sector 2, and 5 ± 1 mm in sector 1. The impacted canines having the linear distance from the midline in the range of 10-15 mm were the most frequent and then in the range of 5 – 10 mm, followed by the range 15 – 20, 20 – 30 and least in the range of 1 – 5 mm (Figure 3). The impacted canines having the linear distance from the occlusal plane in the range of 5-10 mm were the most frequent and then in the range of 10 – 15 mm, followed by the range 1 – 5 mm and equal in number in the ranges 15 – 20 mm and 20 -25 mm (Figure 4).
3. Sagittal view – The angle formed by the line drawn with respect to the occlusal reference lines and the line drawn from the long axis of the impacted canine showed the favorability of eruption of the impacted canine. Ericson and Kurol suggested 250 as the standard angle. The prognosis of the impacted teeth is higher as this angle increases and smaller as the angle decreases.

### Observations drawn from the OPG

The observations drawn from the OPG were the sector method, “α” angle, and linear distance “d”.

When using the sector method, the lines drawn from the long axis of the incisors, canines and first premolar give three sectors:

• Sector 1 – There were 3 (12.5%) teeth in this sector, showing that the prognosis of the impacted canine is poor.
• Sector 2 – There were 8 (33.33%) teeth in this sector, showing that the prognosis of the impacted canine could be favorable but with a risk of resorbing the adjacent root of the lateral incisor.
• Sector 3 – There were 11 (45.8%) teeth in this sector, showing that the prognosis of the impacted canine was good. In this sector, the chance of resorption of the adjacent roots of the incisor was the smallest, as there was no approximation to them.

Transposition – There were 2 (8.33%) teeth with transposition between the two premolars. The impacted canines in this sector fall behind sector 3, giving a questionable prognosis.

The α angle – This angle suggested the favorability of the impacted canine. As this angle increases, keeping 250 as the standard angle suggested by Ericson and Kurol, the prognosis of the impacted teeth was more and less as the angle decreases. The risk of resorption of the root of the lateral incisor increased by 37.5% if the cusp of the canine belonged to sector 1 or 2, and the α angle was higher than 250.

The distance from the occlusal plane to the cusp tip of the impacted canine is almost the same in all cases in both OPG and CT. This was confirmed by Pearson's correlation test, where R2, the coefficient of correlation, was 0.866 (correlation is significant at the level of 0.01). This showed that both CT and OPG had almost the same accuracy in linear measurement from the occlusal plane to the canine cusp (Table 5 AB).

**Table 5:** Correlations between CT and OPG observations. A. Descriptive Statistics.

	Mean	Std. Deviation	N
**Linear dist-d occlusal_OPG**	11.6250	5.95499	24
**Linear dist-m midline_OPG**	14.1250	7.30879	24
**Linear dist-d occlusal_CT**	10.0250	4.41472	24
**Linear dist-m midline_CT**	12.0792	5.35480	24

B. Correlations					
		Linear dist-d occlusal_OPG	Linear dist-m midline_OPG	Linear dist-d occlusal_CT	Linear dist-m midline_CT
Linear dist-d occlusal_OPG	Pearson Correlation	1	-.267	.866(**)	-.298
	Sig. (two-tailed)		.208	.000	.157
N	24	24	24	24
Linear dist-m midline_OPG	Pearson Correlation	-.267	1	-.344	.835(**)
	Sig. (two-tailed)	.208		.100	.000
	N	24	24	24	24
Linear dist-d occlusal_CT	Pearson Correlation	.866(**)	-.344	1	-.308
	Sig. (two-tailed)	.000	.100		.143
	N	24	24	24	24
Linear dist-m midline_CT	Pearson Correlation	-.298	.835(**)	-.308	1
	Sig. (two-tailed)	.157	.000	.143	
	N	24	24	24	24

Note: ** Correlation is significant at the 0.01 level (2-tailed).

**Figure 5:**
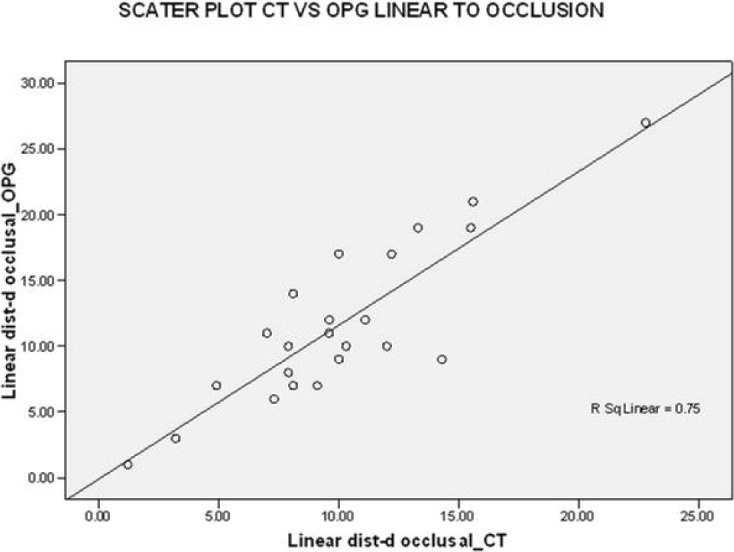
Scatter plot CT vs. OPG, linear to occlusal.

These values of the linear distance from the occlusal plane were correlated on a scattered graph. The linear values of OPG on the x-axis and the linear values of CT on the y-axis represented an R-squared linear of 0.75, indicating that the linear values have a good correlation with each other and that the CT had an accuracy of 0.25 (25%) more than OPG (Figure 5).

**Figure 6:**
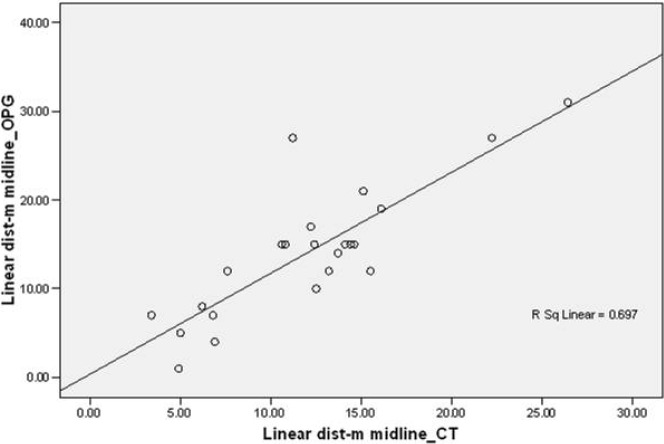
Scatter plot CT vs. OPG, linear midline.

**Figure 7:**
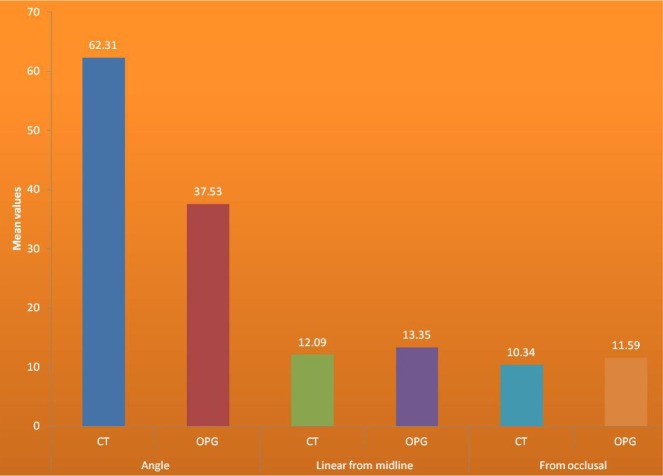
CT and OPG correlation.

In both OPG and CT, the linear distance from the midline plane to the cusp tip of the impacted canine was almost the same in all 24 teeth. This was confirmed by Pearson's correlation test, where R2, the coefficient of correlation, was 0.835 (coefficient is significant at the level of 0.01). This showed that both CT and OPG had almost the same accuracy in linear measurement from the midline plane to the canine cusp. These values of the linear distance from the midline plane were correlated on a scattered graph. The linear values of OPG on the x-axis and the linear values of CT on the y-axis represented an R-squared linear of 0.697, indicating that the linear values had a good correlation with each other and that the CT had an accuracy of 0.31 (31%) more than OPG (Figures 6 and 7).

## Discussion

The causes for retarded eruption of teeth or impacted teeth may be either generalized like endocrine deficiencies, febrile diseases or localized like tooth size-arch length discrepancies, prolonged retention or an early loss of the deciduous canine, ankylosis, idiopathic condition with no apparent cause, absence of the maxillary lateral incisor and others [4-7]. Al-Zoubi H et al. showed that maxillary canines are impacted more frequently after third molars; the emphasis of this study was restricted to them. There is a need for proper localization of the impacted tooth for both access for the surgical approach, and also for the proper direction of the application of orthodontic forces [8].

It is difficult to diagnose and plan the treatment with conventional radiographic methods due to the superimposition of structures. The advantage of CT is that it provides good contrast and reduces blurring and overlapping of adjacent teeth and accurately locates the nearby anatomical structures, recognizing any root resorption in the adjacent teeth [2].

Ericson et al. reported that canines are visualized about 50% better by CT scanning than by conventional intraoral X-rays [9]. Faber et al. showed that diagnosing impacted canines by CT gives better interpretation than conventional radiography, particularly when using 3D reconstructions [10].

We carried out this study to compare CT and OPG for precision in diagnosing the location of the impacted canine and aid the orthodontic management. In this study, only one case involved the resorption of the first premolar, which might be due to the proximity of the impacted canine, suggesting that resorption of the first premolar is unusual, results that were similar to the ones obtained by Walker et al. [1].

The proximity with the central incisor with buccally placed impacted canines was 3 (12.5%), and with lateral incisor were 3 (12.5%) as well. The proximity of the central incisor with a palatally impacted canine was found in 4 (16.6%) cases and 6 (25%) in the case of the lateral incisor, whereas Walker et al. found that the impacted canine was in contact with the lateral incisor in 63.0% cases and with the central incisor in 18.5% cases [1]. In contrast to Walker et al., who found that most (92.6%) of the 27 impactions were palatally placed, we found more buccally placed canines than palatally placed ones [1].

Krennmair et al. showed that CT provides information that is not revealed by traditional radiographic analysis and hence indicated in complicated cases of impacted canines.2

Ehsan Eslami et al. concluded that CBCT is more effective than conventional radiographs in evaluating cases that are difficult to diagnose [11]. Pico et al. found that OPG and CBCT revealed better localization of impacted canines and resorption of adjacent teeth than conventional radiographs [12]. Grisar K et al. also found that 3D enabled imaging systems like CBCT gave better imaging regarding the localization of impacted canines than conventional radiographs [6].

Conventional radiographs have the major disadvantage of superimposition of structures on the film, which makes it very difficult to figure out the details. This, in turn, makes the diagnosis and treatment planning improper as orthodontists require accurate information in order to plan and apply the mechanotherapy strategy. In cases of localization of impacted canines, orthodontists need the exact information about the precise site of the crown and root apex and orientation of the long axis, the closeness of the impacted tooth to the adjacent teeth roots, presence or absence of any pathology, like supernumerary teeth, apical granulomas, or cysts. In recent years, advanced three-dimensional techniques like CT is on the rise as they provide excellent tissue contrast and also reduce any blurring or overlapping of adjacent structures or teeth. The major disadvantage of these latest 3D techniques like CT or cone-beam computed tomography is given by a cost-benefit analysis and expertise in reading the images [11-13].

The limitations of our study involve the small sample size and that only the precise localization of the impacted canine was given greater importance than the difference of radiation between the conventional radiographs and CT scans.

This study helps in assessing the prevalence, proximity, prognosis, approximation, resorption of the adjacent teeth roots, and the linear distance from the midline and occlusal surfaces for the orthodontic assessment of the treatment of impacted maxillary canines. This stressed the importance of CT three-dimensional imaging over the conventional standard OPG and other radiographic films and procedures. This study corroborates the claims made regarding the superiority of CT over OPG in localizing impacted maxillary canines by revealing an increased accuracy in about 31% cases.

## Conclusion

Impacted tooth localization and the inclination of its long axis influence its prognosis, duration of treatment, and the difficulty of surgery. We found that CT provides more reliable information than OPG in features like the exact location of the canine, whether it is buccal or palatal, and whether it is resorbing any adjacent roots. With the added advantage of CT and its three-dimensional viewing capabilities in contrast to OPG, it shows an exact approximation of the impacted canine with the adjacent structures, thereby helping the orthodontist to decide whether to expose, extract or surgically remove it.

## Conflict of Interest

The authors confirm that there are no conflicts of interest.
